# ASCs derived from burn patients are more prone to increased oxidative metabolism and reactive oxygen species upon passaging

**DOI:** 10.1186/s13287-021-02327-4

**Published:** 2021-05-06

**Authors:** David M. Burmeister, Grace Chu-Yuan Chu, Tony Chao, Tiffany C. Heard, Belinda I. Gómez, Linda E. Sousse, Shanmugasundaram Natesan, Robert J. Christy

**Affiliations:** 1 Department of Medicine, Uniformed Services University of the Health Sciences, 4301 Jones Bridge Road, Bethesda, MD 20814 USA; 2 United States Army Institute of Surgical Research, JBSA Fort Sam Houston, 3698 Chambers Pass, San Antonio, TX USA; 3 University of Texas Medical Branch, 301 University Blvd, Galveston, TX 77555 USA

**Keywords:** Adipose stem cells, Mitochondria, ROS, Burn, Respirometry, Glycolysis, Oxidative phosphorylation

## Abstract

**Background:**

Patients with severe burn injury (over 20% of the total body surface area) experience profound hypermetabolism which significantly prolongs wound healing. Adipose-derived stem cells (ASCs) have been proposed as an attractive solution for treating burn wounds, including the potential for autologous ASC expansion. While subcutaneous adipocytes display an altered metabolic profile post-burn, it is not known if this is the case with the stem cells associated with the adipose tissue.

**Methods:**

ASCs were isolated from discarded burn skin of severely injured human subjects (BH, *n* = 6) and unburned subcutaneous adipose tissue of patients undergoing elective abdominoplasty (UH, *n* = 6) and were analyzed at passages 2, 4, and 6. Flow cytometry was used to quantify ASC cell surface markers CD90, CD105, and CD73. Mitochondrial abundance and reactive oxygen species (ROS) production were determined with MitoTracker Green and MitoSOX Red, respectively, while JC-10 Mitochondrial Membrane Potential Assays were also performed. Mitochondrial respiration and glycolysis were analyzed with a high-resolution respirometer (Seahorse XFe24 Analyzer).

**Results:**

There was no difference in age between BH and UH (34 ± 6 and 41 ± 4 years, respectively, *P* = 0.49). While passage 2 ASCs had lower ASC marker expression than subsequent passages, there were no significant differences in the expression between BH and UH ASCs. Similarly, no differences in mitochondrial abundance or membrane potential were found amongst passages or groups. Two-way ANOVA showed a significant effect (*P* < 0.01) of passaging on mitochondrial ROS production, with increased ROS in BH ASCs at later passages. Oxidative phosphorylation capacities (leak and maximal respiration) increased significantly in BH ASCs (*P* = 0.035) but not UH ASCs. On the contrary, basal glycolysis significantly decreased in BH ASCs (*P* = 0.011) with subsequent passaging, but not UH ASCs.

**Conclusions:**

In conclusion, ASCs from burned individuals become increasingly oxidative and less glycolytic upon passaging when compared to ASCs from unburned patients. This increase in oxidative capacities was associated with ROS production in later passages. While the autologous expansion of ASCs holds great promise for treating burned patients with limited donor sites, the potential negative consequences of using them require further investigation.

## Background

Severely burned patients (e.g., > 20% total body surface area (TBSA)) undergo a state of prolonged hyperinflammation and hypermetabolism lasting years post-burn that impairs wound healing [1]. If left unchecked, these burn-induced disturbances may also lead to additional deleterious co-morbidities, such as sepsis, multiple organ dysfunction, and death [2–5]. Moreover, it has been shown that the rate of wound healing differs in survivors versus those that succumb to their injury [6]. Even with increased survival associated with improved care of burned patients, there is often the need for multiple surgeries due to inadequate outcomes and unsuccesful wound healing. This is especially the case with extensive burns where there are limited donor sites for autografting during surgery.

In these scenarios, the potential for using tissue engineering strategies for coverage of excised burn skin has been of great interest. While a recently FDA-approved strategy uses autologous cells [7], exploration of novel allogeneic therapies is an attractive solution to treat burned patients [8]. To this end, adipose-derived stem cells (ASCs) are a type of mesenchymal stem cell that possess immunosuppressive activity, making their allogeneic use possible [9, 10]. In fact, ASCs have been suggested for use in COVID-19 symptoms due to their anti-inflammatory activity [11]. ASCs have other properties that also render them promising, including their angiogenic activity [12] and ease of isolation, including debrided burn tissue [13, 14]. Pluripotent ASCs possess differentiation capabilities that accelerate wound healing [15] and can even stimulate closure of hard-to-treat chronic wounds by growth factor secretion [16]. Evidence to support the therapeutic potential of ASCs to improve wound healing after thermal burns were previously demonstrated in small and large animal models [17–19].

In addition to allogeneic strategies with ASCs, the presence of these cells in medical waste after burn debridement opens the avenue for autologous treatments or tissue engineering strategies [20]. This requires ASC expansion in culture which importantly does not stimulate allogeneic T cells and thus maintains immunocompatibility [9]. However, placing ASCs in artificial culture conditions requires precious time and may affect their metabolic profile. While culture conditions seem to affect ASC phenotype [21], much less is known about how burn-induced metabolic alterations affect ASC expansion. It is known that severe burn trauma alters the metabolic profile of subcutaneous adipose tissue in both animals and humans [22, 23]. Specifically, adipose tissue browning occurs post-burn, wherein increased metabolic activity in adipose tissue contributes to the burn-related hypermetabolism. Whether these changes are reflective of alterations in the stem cells within adipose tissues is currently unknown. The purpose of this study was to determine the bioenergetic capacity by examining mitochondrial respiration and glycolysis of cultured ASCs from burned and non-burned human patients. We hypothesized that the systemic alterations (e.g., inflammation) present in burned patients would result in hypermetabolic ASCs in culture, which was assessed with high-resolution respirometry via a Seahorse Analyzer.

## Methods

### Tissue culture of adipose-derived stem cells

This study was conducted under a protocol reviewed and approved by the US Army Medical Research and Development Command Institutional Review Board and in accordance with the approved protocol. Burn patients undergoing wound excision and abdominoplasty patients undergoing elective surgery have consented to this study through an approved IRB protocol. ASCs were isolated from severely burned patients (BH, *n* = 6) and unburned abdominoplasty patients (UH, *n* = 6) as previously described [14]. ASCs were expanded in Mesenpro RS™ growth media containing growth supplements, 200 mM l-glutamine, and antibiotic-antimycotic which were all provided by Gibco (Thermo Fisher Scientific, Grand Island, NY). The cells were incubated in 37 °C and 5% CO_2_, and upon reaching 80% confluency, they were trypsinized and harvested for analysis at passages 2, 4, and 6 which have been previously characterized for these cells [21]. Total cell count was determined with the trypan blue exclusion method and counted using an automated cell counter (Countess, Invitrogen™, Fisher Scientific, Grand Island, NY).

### ASC identification with flow cytometry

Although these cells have been previously characterized for surface marker expression [14], flow cytometry was used to determine the positive expression of ASC surface proteins CD90, CD105, and CD73 (BD Biosciences, Franklin Lakes, NJ). Cells were stained according to the manufacturer’s instructions. Briefly, 100 μl of cell suspension containing 250,000 cells was incubated with a 5-μl Fc block (BD Biosciences, Franklin Lakes, NJ). Then, 15 μl of positive stem cell markers was added to the cells and incubated for 30 min in a dark incubator at 37 °C and 5% CO_2_. Afterwards, cells were centrifuged, washed with Hanks’ Balanced Salt Solution (HBSS), centrifuged again, and resuspended with 500 μl of HBSS for flow cytometry analysis.

Mitochondrial abundance was determined with MitoTracker Green (Invitrogen™, Fisher Scientific, Grand Island, NY) and mitochondrial ROS production with MitoSOX Red (Invitrogen™, Fisher Scientific, Grand Island, NY). The stock solution of the dyes was prepared which were further diluted for a working concentration according to the manufacturer’s recommendation. Similarly to the ASC markers, 100 μl of cell suspension containing approximately 250,000 cells was incubated with MitoTracker Green at 200 nM concentration and MitoSOX Red at 5 μM concentration. After incubation with the respective dye, the samples were analyzed with the BD FACSCanto II system (BD Biosciences, Franklin Lakes, NJ). This reaction was also carried out in 6-well plates for fluorescent imaging of live cells counterstained with 4′,6-diamidino-2-phenylindole (DAPI) using a Zeiss Observer D1 inverted microscope (Carl Zeiss, Thornwood, NY).

### Mitochondria membrane potential

The JC-10 Mitochondrial Membrane Potential Assay (Sigma-Aldrich, St. Louis, MO) was used to determine mitochondrial membrane integrity. ASCs were plated (20,000 cells/well) in a microplate and incubated in a dark incubator at 37 °C and 5% CO_2_ overnight. ASCs were dyed with JC-10 solution according to the manufacturer’s instructions with parallel groups treated with vehicle only (negative control) and trifluoromethoxy carbonylcyanide phenylhydrazone (FCCP): a protonophore and uncoupler of oxidative phosphorylation which depolarizes the mitochondrial membrane potential and thus serves as a positive control. After the treatment and incubation period, the plate was read with a plate reader (Molecular Devices, Sunnyvale, CA) to measure the fluorescence intensity at *λ*ex = 490/*λ*em = 525 nm and *λ*ex = 540/*λ*em = 590 nm for ratio analysis. The ratio of red/green fluorescence intensity is used to determine the mitochondrial membrane potential.

### Mitochondrial respiration and glycolysis with Seahorse bioanalyzer

Cell Mito Stress Tests and the Glycolytic Rate Assays were performed using a Seahorse XFe24 Analyzer (Agilent, Santa Clara, CA). An equal number of cells (20,000 ASCs/well) were seeded in Seahorse cell culture microplates (Agilent, Santa Clara, CA) 1 day prior to the experiments and incubated in MesenPro™ growth media at 37 °C and 5% CO_2_ overnight. The sensor cartridges were hydrated in Seahorse XF Calibrant Solution (Agilent, Santa Clara, CA) overnight and incubated in a non-CO_2_, 37 °C incubator 1 day before the experiment. XF Assay media was prepared according to the manufacturer’s instructions containing Agilent Seahorse XF Base Medium, 10.5 mM glucose, 1 mM sodium pyruvate, 2 mM l-glutamine, and 5 mM HEPES, and the pH was adjusted to 7.4. An automated protocol for the Cell Mito Stress Test used serial injections of inhibitors and uncouplers to determine the oxygen consumption rate (OCR) in each respiratory state. After a period of equilibration, the basal OCR was determined. Then, 1.0 μM oligomycin, an ATP synthase inhibitor, was added to determine leak respiration that is not coupled to the ATP synthesis. Afterwards, 1.0 μM FCCP was added to determine the maximal respiration of the electron transport chain. Finally, 0.5 μM rotenone/antimycin A was added to inhibit complexes I and II, respectively, to determine the residual respiration indicating proton leak in the mitochondria after inhibition of the electron transport chain.

An automated Glycolytic Rate Assay utilized both the OCR and the extracellular acidification rate (ECAR) to calculate the glycolytic proton efflux rate (PER). After the basal glycolytic PER was measured, the compensatory glycolysis was measured by inhibiting oxidative phosphorylation by injecting the complex I and II inhibitors rotenone and antimycin A (0.5 μM final concentration). Afterwards, 2-deoxy-d-glucose (50 mM final concentration) was added to inhibit hexokinase, thereby inhibiting glycolysis to determine the residual acidification. After glycolytic PER was determined for all states, the residual acidification values were subtracted from basal and compensatory glycolysis to account for acidification from other sources such as the TCA cycle.

### Statistical analysis

Statistical analyses were conducted using the GraphPad Prism software v7 (San Diego, CA). A two-way ANOVA was performed to determine the significance of group and passage, as well as significant interaction. The Shapiro-Wilk tests were performed to determine the normality of each dataset, with post hoc analyses performed as appropriate. One-way ANOVA or Kruskal-Wallis with Dunn’s multiple comparisons were done to determine the differences due to passaging and a Friedman and Mann-Whitney post hoc testing were performed to determine the between-group differences at various passages. Unpaired *t* tests were used for clinical data. Values are presented as mean ± SE. Statistical significance was determined when *P* < 0.05.

## Results

### Patient characteristics

As shown in Table 1, burned patients presented with 56 ± 8% total body surface area burns indicating extensive trauma. Additionally, there was no difference in age between the groups, with burn patients’ (BH) mean age as 34 ± 6 years and unburned patients’ (UH) mean age as 41 ± 4 years (*P* = 0.49).

**Table 1:** Age of both burned (BH) and unburned (UH) patients, with the extent of burn also indicated. Values are expressed as mean ± SEM

	Age (years)	TBSA (%)
BH	34 ± 6	56 ± 8
UH	41 ± 4	–
*P* value	0.49	N/A

### ASC marker expression

**Fig. 1:**
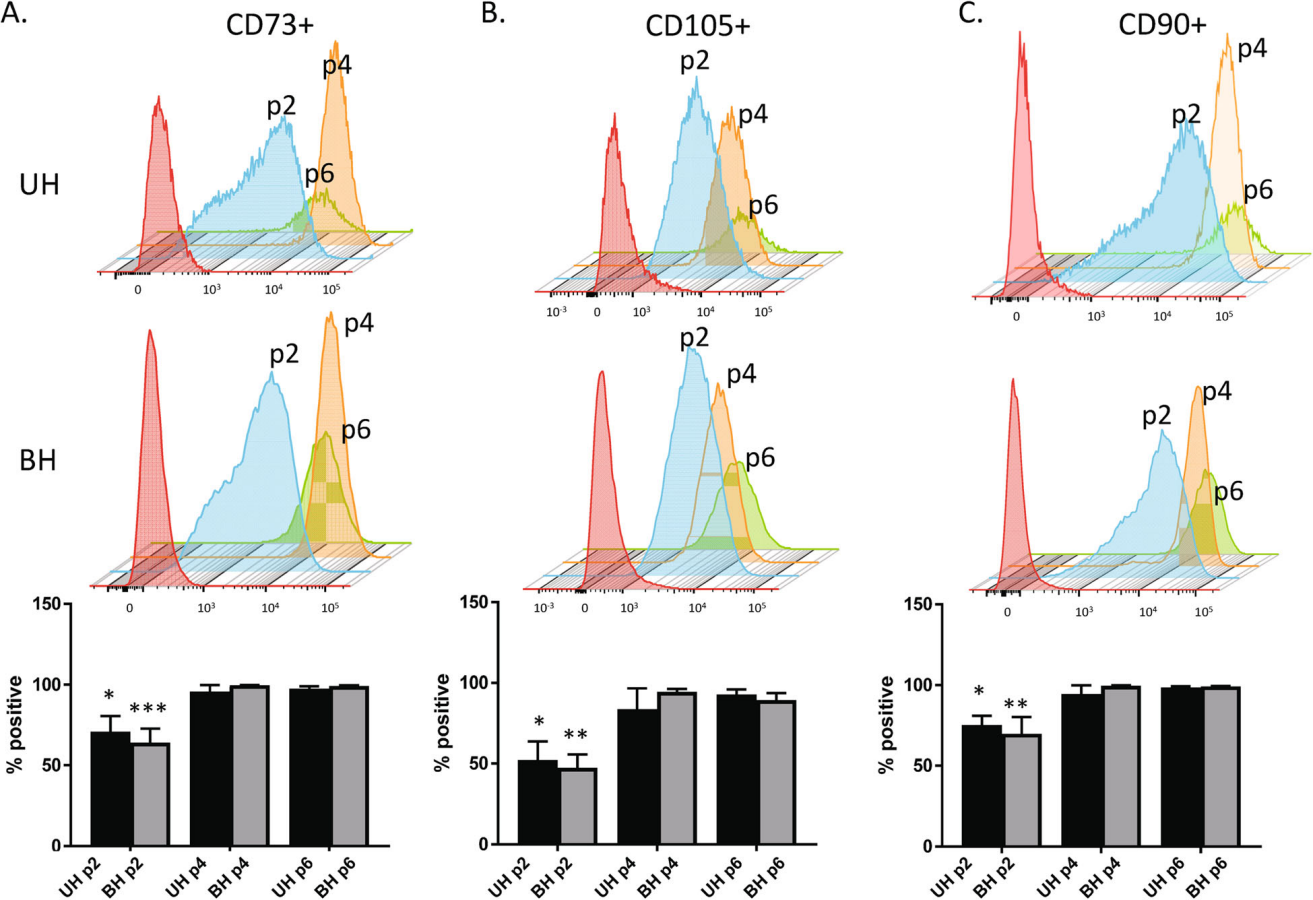
Flow cytometry of ASC markers. Representative flow cytometry readouts for ASC surface marker expression of CD73 (**a**), CD105 (**b**), and CD90 (**c**) for both burned human (BH) and unburned human (UH) patients. The bottom row shows quantification, which shows that while there were no differences in any marker at any passage between BH and UH, there was a significantly lower expression of each marker at passage 2, compared to subsequent passages. Expression of CD73 on UH ASCs was 70.8 ± 9.8, 95.8 ± 3.9, and 97.6 ± 1.3% at passages 2, 4, and 6, respectively, while the expression on BH ASCs was 64.0 ± 8.6, 99.7 ± 0.1, and 99.2 ± 0.4%, respectively. Expression of CD105 on UH ASCs was 52.3 ± 11.7, 83.8 ± 12.9, and 92.9 ± 3.2% at passages 2, 4, and 6, respectively, while the expression on BH ASCs was 47.4 ± 8.2, 94.6 ± 1.8, and 89.5 ± 4.4%, respectively. Expression of CD90 on UH ASCs was 75.3 ± 5.8, 94.4 ± 5.4, and 98.5 ± 0.8% at passages 2, 4, and 6, respectively, while expression on BH ASCs was 69.8 ± 10.5, 99.5 ± 0.3, and 99.2 ± 0.2%, respectively. *P < 0.05, **P < 0.01, ***P<0.001. *N* = 6 patients in BH ASCs and UH ASCs at each passage

Representative flow readouts and quantifications are shown in Fig. 1 for ASC markers CD73 (Fig. 1a), CD105 (Fig. 1b), and CD90 (Fig. 1c). For each of these markers, total percent positive cells were significantly lower at passage 2 compared to the subsequently tested passages for both patient groups, indicating a more heterogeneous population at lower passages. However, no significant differences were found between UH and BH for any of these ASC markers at all passages examined. By passage 4, the overwhelming majority of both BH and UH ASCs were positive for these cell surface markers.

### Mitochondrial characteristics

**Fig. 2:**
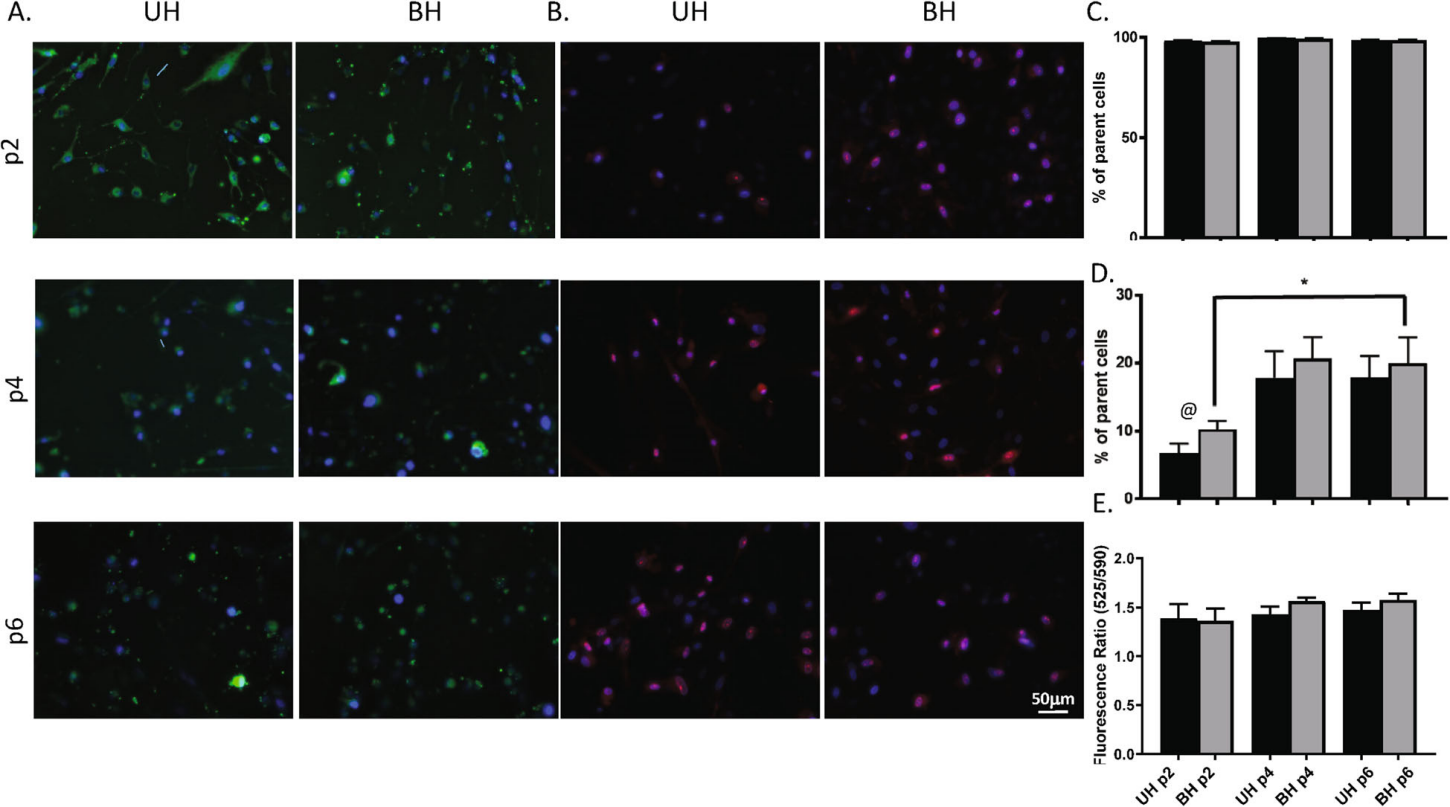
Mitochondrial characteristics of ASCs in culture. Representative images of staining for MitoTracker (**a**) and MitoSOX (**b**) for BH and UH ASCs across passages show relatively consistent quantities of mitochondrial content in green but increasing amount of ROS with passaging in red. Quantification of these fluorescent dyes via flow cytometry shows that nearly all ASCs in both groups stained positive for MitoTracker (**c**), while MitoSOX (**d**) expression increased significantly in passage 6 compared to passage 2 in BH ASCs. *P < 0.05. BH ASCs also displayed more MitoSox-positive cells than UH ASCs at passage 2, ^@^*P* < 0.05. **e** The mitochondrial membrane potential dye, JC-10, was not different between the groups or passages. *N* = 6 patients in BH ASCs and UH ASCs at each passage

Representative images for MitoTracker and mitochondrial ROS are shown in Fig. 2a and b, with flow cytometry quantification shown in Fig. 2c and d, respectively. MitoTracker assays reveal abundant mitochondria in both BH and UH ASCs, with all passages showing at least 98% positive cells via flow cytometry (Fig. 2c). Two-way repeated measures ANOVA revealed a significant effect of passage (*P* = 0.0216) wherein ASCs from both groups produced more ROS in later passages. However, the difference due to burn did not reach statistical significance (*P* = 0.0641). When the post hoc Mann-Whitney tests were performed, there were significantly higher MitoSOX positive ASCs from BH patients (10.27 ± 1.25%) at passage 2 compared to UH ASCs (6.76 ± 1.42%; *P* = 0.047). This was not true for passage 4 (*P* = 0.485) or passage 6 (*P* = 0.818). Two-way ANOVA of the JC-10 assay revealed no effect of passage (*P* = 0.273) or burn (*P* = 0.368) on mitochondrial membrane potential (Fig. 2e).

### ASC oxidative phosphorylation

**Fig. 3:**
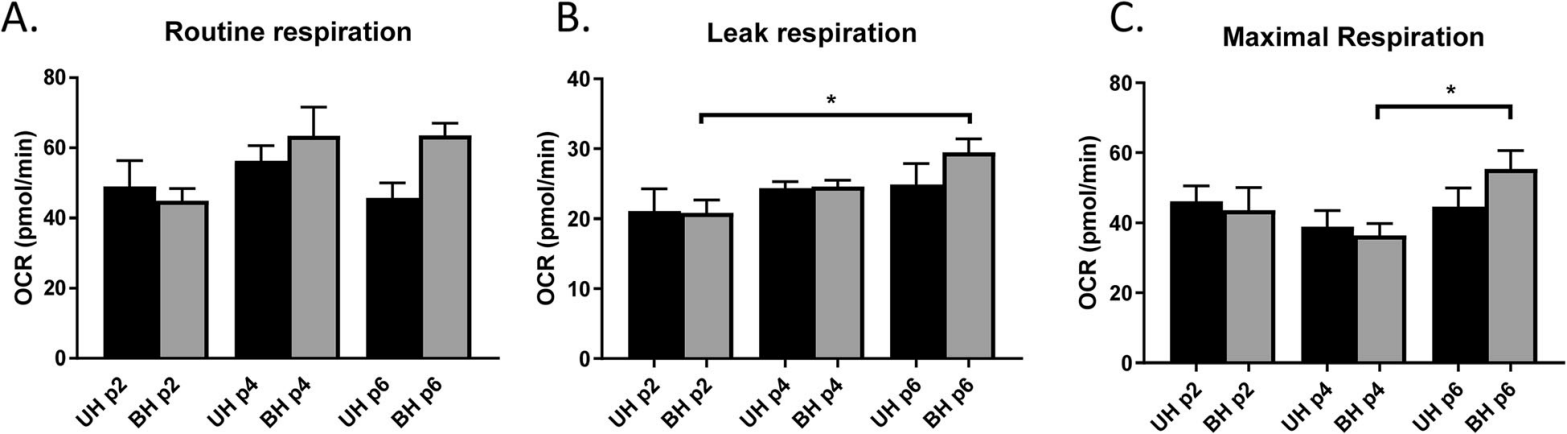
Oxidative phosphorylation capacity of ASCs in culture. High-resolution respirometry revealed a non-significant increase in routine (**a**) respiration across passages. Alternatively, both leak (**b**) and maximal (**c**) respiration were significantly higher in BH ASCs when taken to passage 6 as compared to passages 4 and 2, respectively. *P < 0.05. *N* = 6 patients in BH ASCs and UH ASCs at each passage. OCR, oxygen consumption rate

To determine whether this increase in ROS was associated with higher oxidative phosphorylation capacities, a Mito Stress Test was performed (Fig. 3). Two-way ANOVA revealed a significant effect of burn (*P =* 0.0383) but not passaging (*P* = 0.1396) on routine respiration (Fig. 3a). Post hoc testing, however, did not show a significant difference between BH and UH ASCs, even at passage 6, which showed the biggest difference (*P* = 0.0853). Two-way ANOVA analysis of both leak (Fig. 3b) and maximal respiration (Fig. 3c) revealed a trend for an effect of passage, which did not quite obtain significance (*P* = 0.0536 and *P* = 0.0620, respectively). However, post hoc testing corrected for multiple comparisons (Sidak’s tests) revealed that BH ASCs displayed higher oxidative phosphorylation capacities at passage 6 compared to earlier passages (p2 for leak, *P* = 0.047, p4 for maximal, *P* = 0.0349), which was not true for UH ASCs.

### Glycolysis

**Fig. 4:**
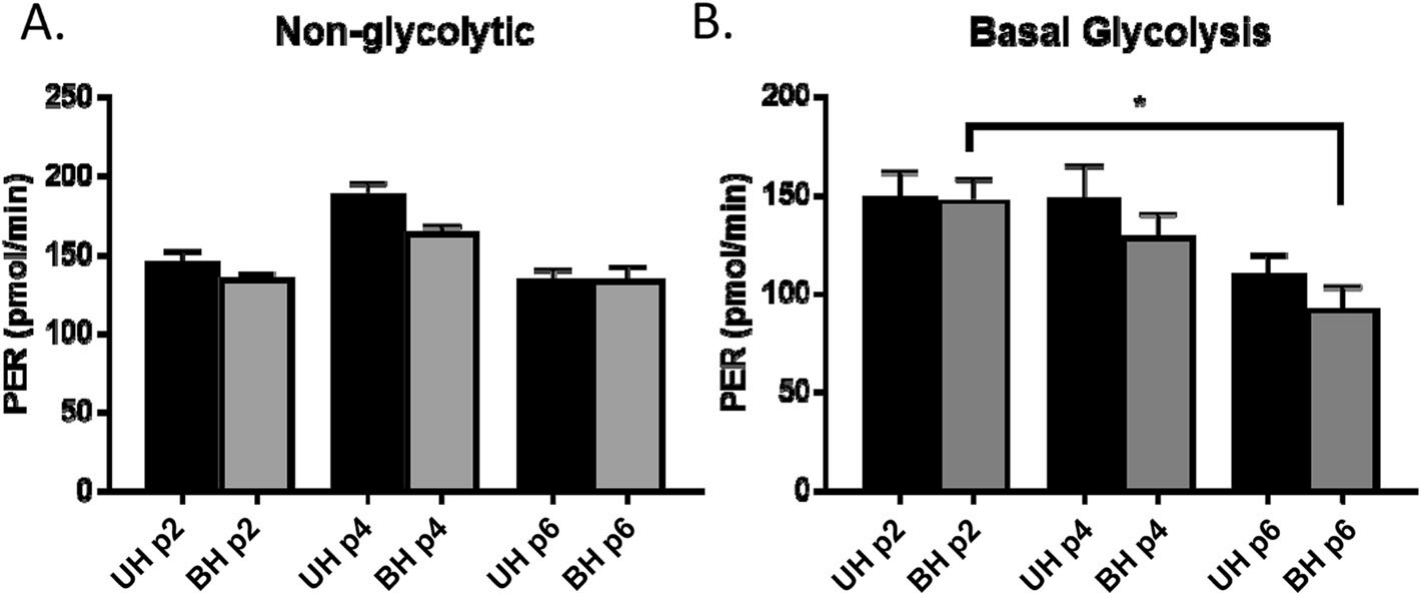
Glycolytic capacity of ASCs in culture. While no differences were found between the groups or across passage for the non-glycolytic PER (**a**), a significant decrease in the basal glycolytic rate (**b**) was found when comparing passage 6 to passage 2 for BH ASCs, but not for UH ASCs. *P < 0.05. *N* = 6 patients in BH ASCs and UH ASCs at each passage. PER, glycolytic proton efflux rate

**Fig. 5:**
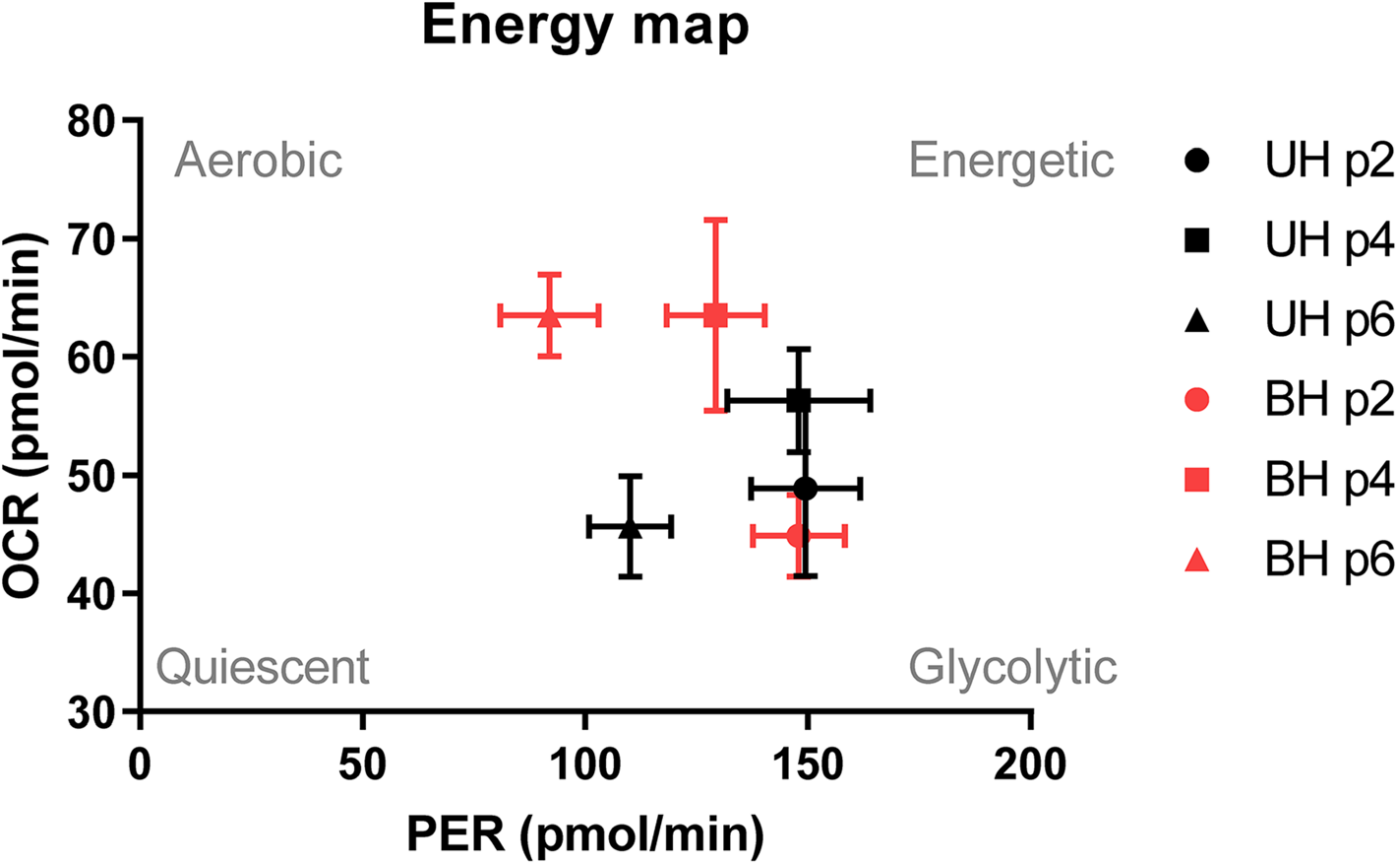
Energy map of BH and UH ASCs across passages. Plotting the oxygen consumption rate (OCR) versus the glycolytic proton efflux (PER) reveals that, when compared to UH ASCs, BH ASCs become more aerobic and less glycolytic as they are passaged

To determine whether this increase in oxidative phosphorylation occurred with concomitant changes in glycolysis, a Glycolytic Rate Assay was performed (Fig. 4). Basal glycolysis was corrected for by subtracting pH changes that were not due to glycolysis. Two-way ANOVA revealed a significant effect of passaging on ASC glycolysis (*P* = 0.0019). However, post hoc testing only revealed that BH ASCs displayed reduced glycolysis at passage 6 when compared to passage 2 (*P* = 0.011). Another way to visualize the overall metabolic activity of these cells is to plot glycolysis against oxidative phosphorylative capacities (Fig. 5). Doing so reveals that, when compared to UH ASCs, BH ASCs become more aerobic and less glycolytic with subsequent passaging.

## Discussion

Adipose stem cells (ASCs) hold great promise for tissue engineering strategies that replace damaged or missing tissue such as after an extensive burn. Specifically for burned patients, autologous strategies are of great interest since medical waste tissue generated during excision and grafting contains viable ASCs [14]. This strategy would benefit from the expansion of these cells in culture for increased coverage area. However, it is not known whether the significant systemic aberrations that occur after burns negatively affect their expansion potential. While it has been shown that culture conditions can alter their expansion [21], the current study was undertaken to see if their metabolic phenotype was altered. The salient findings include that ASCs from burned patients are relatively unchanged from other patients from a bioenergetics perspective, but that their oxidative phosphorylation capacities increase with passaging, as does the levels of mitochondrial ROS.

It has been recently shown that the sustained hypermetabolism seen in burned patients is, in part, due to the browning of the subcutaneous adipose tissue [23]. Both in the acute and chronic time frames, this tissue displays increased oxidative phosphorylation, mitochondrial mass, and mitochondrial uncoupling that is associated with higher expression of the adipose-specific uncoupling protein 1 (UCP1) [24, 25]. Interestingly, ASCs from burned patients did not display increased oxygen consumption in the basal or the leak states when compared to ASCs from non-burned patients. Previously, it has been shown that IL-6 derived from brown adipose tissue is important for glucose homeostasis, and perhaps the lack of inflammatory signals (not measured herein) in the tissue used in the current study may have precluded metabolic differences in ASCs [26].

We also did not see any appreciable differences in MitoTracker staining which was true even at passage 2, where flow cytometry data indicated a more heterogeneous population of cells. This was seen as higher side scatter in those cells (P2, Fig. 1) and can also be seen in the MitoTracker Staining (Fig. 2). The reduction in heterogeneity across passaging in these cells has been seen previously [14]. This is also in line with other studies showing the higher doubling rate of ASCs causes the proportion of ASCs to increase upon passaging [27–30]. Regardless, we cannot rule out the effects of artificial culture conditions affecting (even normalizing) the metabolic activity of these ASCs. While fresh tissue (i.e., cells derived from adipose without passaging) from the patients included in this study was not analyzed for metabolic endpoints, this would be challenging due to the fact that isolation of ASCs is often dependent on their expansion to avoid a heterogeneous population of cells (i.e., stromal vascular fractions or SVF). Similarly, the time from isolation to analysis, and subsequent passaging could be a viable explanation for the lack of metabolic alterations seen in the ASCs reported herein.

Alternatively, it is possible that ASCs do not contribute to metabolic changes seen in adipose tissue post-burn. Subcutaneous browning has been proposed to be driven by inflammatory processes such as NOD-, LRR-, and pyrin domain-containing protein 3 (NLRP3) inflammasomes [31] and macrophage polarization [32]. Systemic inflammation in burned patients is a serious comorbidity proportional to the TBSA involved [33] and does increase the bioenergetics capacity of circulating lymphocytes [34]. Moreover, free radical activity has been tied to survival in burn patients [35]. However, it has been shown that the inflammation associated with the SVF in burn tissue is not due to the ASCs, but rather the other stromal cells present [36]. Thus, taken together with the lack of differences in metabolic phenotype seen herein, it is likely that both burn-induced inflammation and hypermetabolism in adipose tissue originate from adipocytes or other stromal cells as opposed to ASCs.

In this regard, various strategies using adipose tissue have been used for regenerative medicine and wound healing purposes. For example, not only isolated ASCs, but also the SVF, has been shown to enhance wound healing and has been used in burn patients [37]. Moreover, the cells within SVF have been utilized in tissue engineering strategies in combination with, for example, extracellular matrices and platelet-rich plasma [38–42], which can affect the differentiation potential of ASCs [43]. Another consideration is that the strategy used to isolate ASCs or SVF (for example, enzymatic vs. mechanical digestion) might also alter their properties [44, 45]. Taken one step further, whole-tissue fat grafting (to include adipocytes) has potential use in burns and scarring [39, 46]. On the other extreme, the regenerative properties of ASCs may be further selected for by harnessing the exosomes released from them, or even the contents (e.g., microRNAs) of those exosomes [47, 48]. While the exact isolation method and the subset of adipose constituents may result in different metabolic phenotypes, we chose to concentrate on cultured cells in order to aim for a homogenous population of cells, which may have obscured differences in the metabolism.

The basal respiration values reported herein are similar to previously reported values from ASCs isolated from the abdominal fat [49]. While the anatomical location has not been shown to drastically affect the metabolic profile of ASCs, obesity has been shown to negatively affect the oxygen consumption rate in ASCs [50]. There are methodological differences that may explain why we did not find a similar observation, since we utilized the proton efflux rate assay as opposed to using the extracellular acidification rate found with the mitochondrial stress assay. This assay is a robust measure of glycolysis that uses 2-deoxy-d-glucose, which inhibits the phosphorylation of glucose by hexokinase, to adjust for hydrogen protons not created by a glycolytic source. However, the mitochondrial assay media is not able to replicate the environment or substrate availability ASCs are exposed to in situ.

Interestingly, the reduced oxygen consumption rate from obese ASCs reported by Perez et al. was only seen when glucose was the carbon source, and a switch to short-chain fatty acids (SCFAs) reversed this effect. We did not examine the presence of SCFAs in the current study, but it has been shown that intestinal epithelial cells (which utilize SCFAs) are impaired in burn injury and work through an inflammasome pathway [51, 52]. While this is an interesting observation, adipose tissue typically uses glucose as an energy source, which likely would be abundant in burn patients that are typically insulin resistant. In fact, whole-body glycolysis and lipolysis rates are increased by 250 and 450%, respectively [53], indicating an overabundance of substrates for the glycolysis and oxidative pathways. Our findings of minimal differences in BH vs UH metabolism suggest that this total body response may be driven by other tissues (e.g., skeletal muscle) or cells within the adipose tissue (adipocytes). As discussed earlier, it is well documented that adipocytes display altered metabolism and that mitochondrial function in the skeletal muscle is also altered by severe burns [54, 55]. Our data suggest that the physiological response to hypermetabolic demand in severe burn recovery is not met by alterations in the ASC metabolism.

The finding of increased ROS with passaging in BH ASCs may have implications for their expansion in tissue engineering strategies. While increased ROS was seen upon passaging in both BH and UH ASCs, it was only significant in the BH cells. The association of increased ROS with higher mitochondrial respirometry has been shown previously in the context of comparing visceral ASCs to subcutaneous ASCs [56]. The expression and production of antioxidants were able to counteract a certain extent of these differences and could be included in the culture expansion of ASCs. In general, the BH ASCs became highly oxidative and less glycolytic, which could inform culture conditions for optimal expansion.

There are several limitations of this study worth mentioning. This represents a retrospective, observational study that was not designed to examine the resting energy expenditure of the patients, nor the metabolic phenotype of isolated cells in fresh tissue. As such, this data is largely de-identified, and associations with clinical outcomes or demographics were not possible. Similarly, we cannot rule out the effect of medications or comorbidities on the glycolytic capacity of these cells. Additionally, the surprising lack of differences between BH and UH cells (despite a relatively high TBSA involvement) dampened enthusiasm for exploring molecular mechanisms of mitochondrial changes, including uncoupling proteins or mitochondrial fusion/fission proteins. Finally, the limited sample size may have contributed to the lack of differences found between the two patient populations. However, the lack of differences emphasizes promise for the strategy of expanding autologous ASCs from burned patients for the purposes of tissue engineering and wound coverage.

## Conclusions

Extensive thermal injury is accompanied by substantial metabolic derangements that begin acutely and persist for years. These same injuries also generate challenges in terms of wound area coverage, which is ideally accomplished with autografting. Tissue engineering strategies to try and cover these open wounds have explored the use of autologous ASCs. To our knowledge, we report the first experience suggesting that the metabolic consequences of burn injury do not negatively affect the bioenergetic capacity of isolated ASCs. However, we show that culture of ASCs from burned patients begin to produce ROS and is accompanied by a more oxidative and less glycolytic phenotype, which has implications for their expansion ex vivo.

## Data Availability

The datasets used and/or analyzed during the current study are available from the corresponding author on reasonable request.

## Abbreviations

ASCs: Adipose-derived stem cells
BH: Burned human patient
UH: Unburned human patient
CD: Cluster of differentiation
ROS: Reactive oxygen species
ANOVA: Analysis of variance
TBSA: Total body surface area
FDA: Food and Drug Administration
IRB: Institutional Review Board
HBSS: Hanks’ Balanced Salt Solution
DAPI: Diamidino-2-phenylindole
FCCP: Trifluoromethoxy carbonylcyanide phenylhydrazone
OCR: Oxygen consumption rate
ECAR: Extracellular acidification rate
PER: Proton efflux rate
TCA: Tricarboxylic acid
UCP1: Uncoupling protein 1
IL: Interleukin
NLRP3: NOD-, LRR-, and pyrin domain-containing protein 3
SCFA: Short-chain fatty acid
